# A new distribution and host record for the rare moth, *Callioratis millari* (Lepidoptera: Geometridae), and some ecological observations

**DOI:** 10.1093/ee/nvae008

**Published:** 2024-02-10

**Authors:** Paul Duvel Janse van Rensburg, Hugo Bezuidenhout, Tommie Steyn, Johnnie van den Berg

**Affiliations:** Unit of Environmental Sciences and Management, North-West University, Private Bag X6001, Potchefstroom 2520, South Africa; Arid Ecosystems Research Unit, Conservation Services, SANParks, P.O. Box 110040, Hadison Park, Kimberley 8306, South Africa; Applied Behavioural Ecology and Ecosystem Research Unit, University of South Africa, Private Bag X6, Florida Campus 1717, South Africa; Terrestrial Systems Unit, Scientific Services, Mpumalanga Tourism and Parks Agency, Lydenburg, South Africa; Unit of Environmental Sciences and Management, North-West University, Private Bag X6001, Potchefstroom 2520, South Africa

**Keywords:** conservation, cycad, threatened, habitat preference, Diptychini

## Abstract

*Callioratis millari* Hampson (Lepidoptera: Geometridae) is a Critically Endangered moth endemic to South Africa. Despite extensive searches, it was previously known only from the Entumeni Nature Reserve in KwaZulu-Natal, where its larvae exclusively feed on the cycad *Stangeria eriopus* (Kunze) Baill (Cycadales: Stangeriaceae). In July 2022, a new population of *C. millari* was discovered in the Kabouga section of Addo Elephant National Park in the Eastern Cape. Larvae of *C. millari* were feeding on the cycad *Encephalartos caffer* (Thunb.) Lehm (Cycadales: Zamiaceae), which also constitutes a new host record. In June 2023, we determined larval incidence and herbivory at this new locality, offering insights into the ecological requirements of *C. millari*. Known *C. millari* localities, although ecologically different, share low altitudes (700–950 m a.s.l.), moderate to high rainfall, and grassy habitats with sparse woody cover. A total of 59 larvae were counted in Kabouga, mostly in the fifth and sixth (final) instars. Herbivory incidence was lower on smaller plants and those covered by other vegetation. The flight period of adult *C. millari* likely occurs between mid-March and April in Kabouga, but further investigation is needed to clarify this. The peak period of larval occurrence in Kabouga occurs during the driest and coldest months of the year (May–July). Considering limited habitat availability, host plant poaching, and the risk of untimely fires, the species should be considered highly threatened. This study adds to our understanding of the biology of *C. millari* and provides information on its ecological requirements and may contribute to making informed management decisions.

## Introduction

*Callioratis millari* Hampson (Lepidoptera: Geometridae) belongs to the subfamily Ennominae in the tribe Diptychini (Murillo-Ramos et al. 2019). Cycad-feeding Diptychini comprises 3 genera: *Callioratis* (6 species), *Zerenopsis* (8 species), and *Veniliodes* (3 species) (Sihvonen et al. 2015). The cycad-feeding moths within the Diptychini differ from other Geometridae in several ways, such as aposematic coloration, diurnal adults, and lek mating behavior (Staude 1996, 2001, Staude et al. 2011, Staude and Sihvonen 2014). For at least their first 3 instars, larvae are specialized to one or a few species of cycad, but larger larvae of several Diptychini species have been recorded to switch to a variety of secondary angiosperm hosts, including species from genera like *Apodytes* (Icacinaceae), *Diospyros* (Ebenaceae), *Carissa* (Apocynaceae), and *Maesa* (Maesaceae) (Staude 1994, 2001, Donaldson and Bösenberg 1995).

The presence of these herbivores raises interesting conservation concerns since they depend on rare plants that occur in fragmented landscapes. Cycads are among the world’s most threatened organisms, facing critical challenges due to habitat loss, poaching, and reproductive failures caused by the extinctions of pollinators (Mankga and Yessoufou 2017, Janse van Rensburg et al. 2023a). Diminishing cycad populations also endanger their specialist herbivores (Oberprieler 1995, Janse van Rensburg et al. 2023a). While some Diptychini, e.g., *Zerenopsis lepida* (Walker), have demonstrated the ability to expand their range to cultivated cycads, which also facilitates expansion to new wild cycad populations with potentially deleterious effects (Janse van Rensburg et al. 2023b), others rely exclusively on a single cycad host or locality. As a result, their survival depends on the health of their host populations and habitat. Therefore, Diptychini species are often of greater conservation concern than their threatened cycad host plants (Bayliss et al. 2009). For example, the threatened *Callioratis grandis* Prout only exists in a particular area of Mount Mulanje in Malawi, making its range much more restricted than its host, *Encephalartos gratus* Prain (Bayliss et al. 2009, Staude et al. 2011).

Notably, *C. millari* is considered the rarest moth in South Africa and is listed as Critically Endangered in the COREL (Custodians of Rare and Endangered Lepidoptera) program of the Lepidopterist’ Society of Africa (Edge 2011, Mecenero et al. 2020). It was originally discovered in an area near Durban, KwaZulu-Natal, which has since succumbed to urban development, leading to the extinction of *C. millari* in that area in 1928. Until recently, it was only known to still occur in one locality, the Entumeni Nature Reserve (Entumeni) in KwaZulu-Natal where it faces severe threats in the form of host plant poaching and untimely fires (Staude 2001, Louw and Armstrong 2018, Terblanche 2018). Despite extensive searches in KwaZulu-Natal grassland areas that contain viable populations of its cycad host, *Stangeria eriopus* (Kunze) Baill, no additional *C. millari* populations have been located (Staude 2001). Larvae feed on leaves of *S. eriopus* that grow in the open grassland patches, but not on *S. eriopus* plants under the surrounding Forest canopy cover (Staude 2001). In captivity, larvae accepted leaves of the cycad *Encephalartos villosus* Lem, but no larvae have been found on *E. villosus*, which occurs only under the Forest canopy cover in Entumeni (Staude 2001). Therefore, *C. millari* appears to have specific vegetation-cum-habitat (habitat) requirements, which may contribute to its rarity (Staude 2001).

**Fig. 1.**
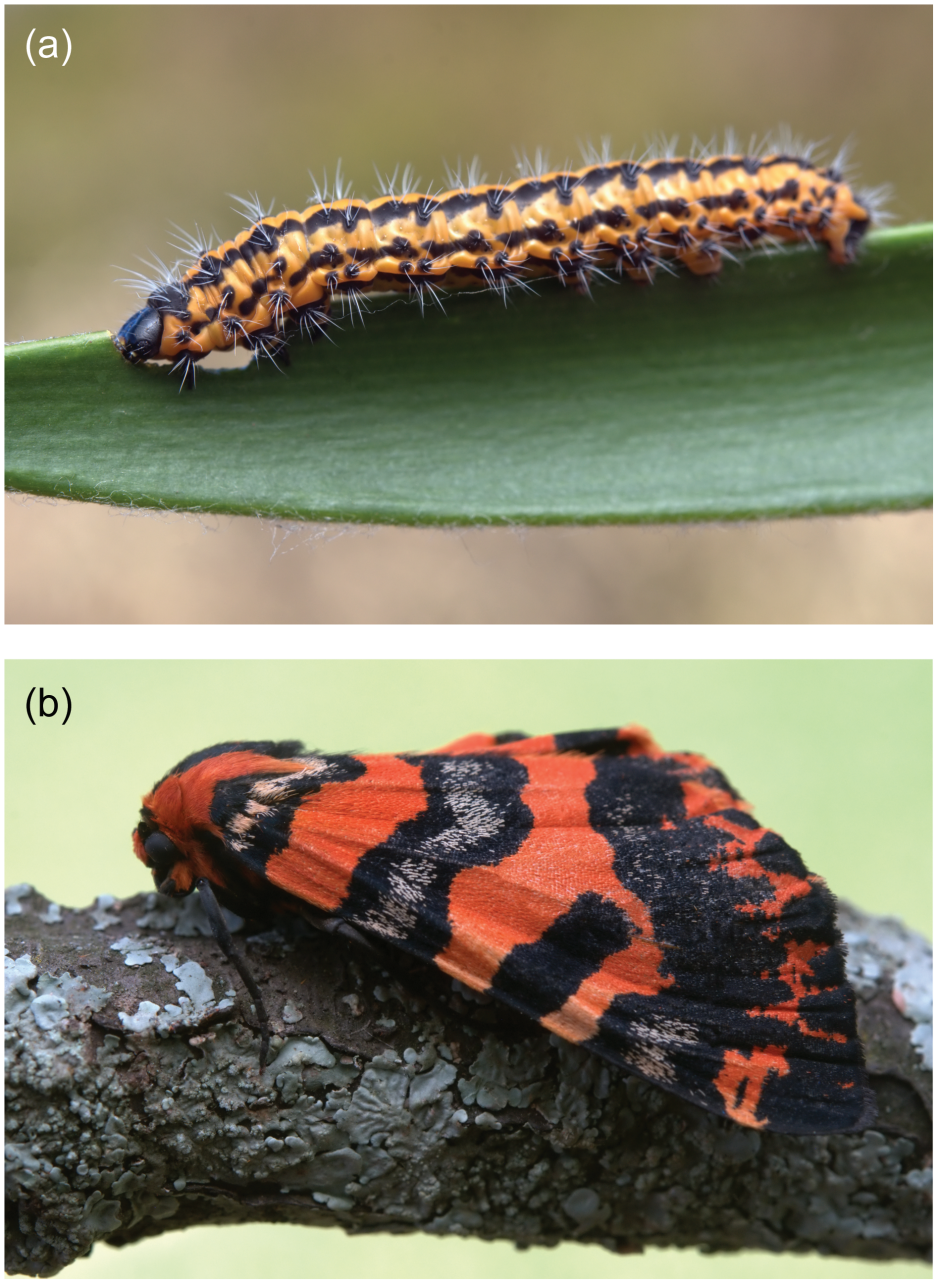
a) *Callioratis millari* larva from the Kabouga section, Addo Elephant National Park; b) adult moth. (Photographs: P.D. Janse van Rensburg).

In this article, we report a new locality and host plant for *C. millari* in the Eastern Cape province. On 11 July 2022, we encountered larvae of an unidentified Diptychini moth feeding on the leaves of the cycad *E. caffer* (Thunb.) Lehm. within a grassy Fynbos patch in the Kabouga section of Addo Elephant National Park (AENP) (Kabouga) (Fig. 1a). Collecting 1 final-instar larva, we observed its pupation shortly thereafter. The resulting adult moth emerged on 28 March 2023 and was later identified as *C. millari* (Fig. 1b). Populations of *Encephalartos lehmannii* Lehm. and *E. longifolius* (Jacq.) Lehm. are present within a 10-km radius of the population of *E. caffer* (Bezuidenhout 2015), but we did not record *C. millari* on these plants. Another Diptychini species, *Z. lepida*, also occurs in Kabouga, and we documented its occurrence on both *E. lehmannii* and *E. longifolius.*

The life-history and habitat requirements of *C. millari* remain poorly understood. In this article, we compare the habitats of the current distribution localities and historical type locality of *C. millari*, aiming to clarify some aspects of its habitat requirements. Additionally, we conducted surveys to assess the incidence of larvae and herbivory by both *C. millari* and *Z. lepida* at Kabouga to obtain preliminary ecological information about these species and to assess potential competition between *C. millari* and *Z. lepida.*

## Materials and Methods

### *Callioratis millari* Localities

**Fig. 2.**
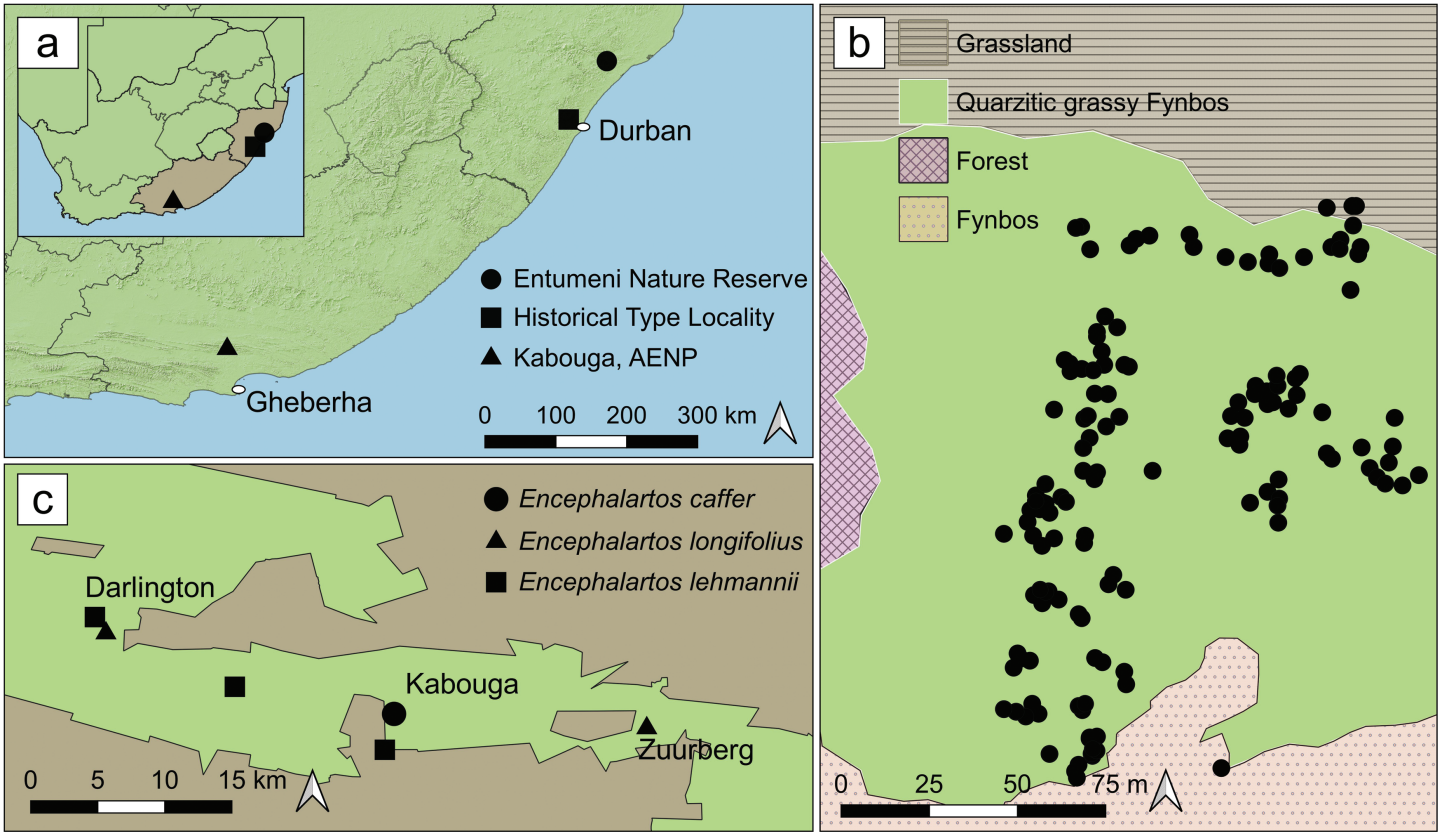
a) Current localities of *Callioratis millari* and the historical type locality; b) map of the *Encephalartos caffer* population in Kabouga in relation to the surrounding plant communities (adapted from Van Wyk et al. 1988); c) approximate localities of *Encephalartos* species in the Darlington, Kabouga, and Zuurberg sections of Addo Elephant National Park.

The historical type locality for *C. millari* was near the railway stations of Kloof, Gillitts, and Hillcrest near Durban (Fig. 2a). However, this area has undergone urban development, leading to the destruction of the original grassland habitat (Staude 2001). It has not been recorded in this area since 1928 (Staude 2001). Following its disappearance in the type locality, *C. millari* was rediscovered in Entumeni in 1997, which lies approximately 120 km further north (Fig. 2a). Adults and larvae of *C. millari* are restricted to 3 open Grassland patches (within 5 km of each other) in and around Entumeni, surrounded by Scarp Forest (Staude 2001, Louw and Armstrong 2018).

The locality of the newly discovered population of *C. millari* is 750 km south of Entumeni and is located in the Kabouga section of the AENP (Fig. 2a). The AENP is divided into 8 sections that are Bird and St Croix Island group, Woody Cape, Colchester, Addo Main Camp, Nyati, Kabouga, Zuurberg, and Darlington. Encompassing approximately 26,000 ha, Kabouga stretches along both sides of Sundays River, extending north to the Darlington section and eastward to the Zuurberg section. Kabouga consists of Sundays River, including the floodplains, Kabouga Thicket, Quarzitic grassy Fynbos, Fynbos, Kabouga Shrubland, Grassland, Forest, and Degraded vegetation plant communities (adapted from Van Wyk et al. 1988). We recorded *C. millari* in the Quarzitic grassy Fynbos, where the larvae were feeding on *E. caffer* individuals (Fig. 2b). The *E. caffer* population is relatively large (>200 individuals) and occurs in a small area (3.9 ha).

The habitat of the *C. millari* population in Kabouga is located on a steep south-facing upper midslope of the Zuurberg mountain at an altitude of ~950 m a.s.l. (Supplementary Table A1; Supplementary Material). The *C. millari* and *E. caffer* populations are strongly associated with the Quarzitic grassy Fynbos plant community and are surrounded by patches of Forest, Grassland on the north-facing midslopes and Fynbos on the relatively lower altitudes (Van Wyk et al. 1988, Bezuidenhout 2015). Unlike other grassy Fynbos areas, the winter months are the driest (Van Wyk et al. 1988). Thunderstorms are common during the summer months, leading to lighting fires. Both Entumeni and the original type locality are situated at slightly lower altitudes (~700 m a.s.l.). The *C. millari* population in Entumeni is found in Moist Coast Hinterland Grassland, surrounded by Scarp Forest (Rutherford et al. 2006). The vegetation consists of tall grassland dominated by *Cymbopogon nardus* (L.) Rendle with a high diversity of flowering forbs (Louw and Armstrong 2018). The original type locality is within the KwaZulu-Natal Sandstone Sourveld (Grassland) and is also surrounded by patches of Scarp Forest (Rutherford et al. 2006). The vegetation would have consisted of a species-rich grassland with scattered shrubs, including Proteaceae species (Rutherford et al. 2006).

The plant communities where *C. millari* occurs in Kabouga and the KwaZulu-Natal localities receive summer rainfall, with Kabouga receiving lower rainfall than the KwaZulu-Natal localities (Rebelo et al. 2006, Rutherford et al. 2006). However, we only recorded *C. millari* along with its host on a steep south-facing upper midslope in Kabouga, where moisture levels are higher (Van Wyk et al. 1988, Rebelo et al. 2006). Frequent mist also contributes to higher moisture levels in the *E. caffer* population in Kabouga compared to lower down in the Zuurberg Valley bottomlands, where it can get dry. The Kwazulu-Natal localities experience slightly higher maximum temperatures, but the minimum temperatures at all the localities can be below 5 °C in the winter months, but compared to the Kabouga locality, frost incidence is less frequent in the KwaZulu-Natal localities. The geology of the *E. caffer* Kabouga locality consists of white quarzitic sandstone and subordinate shale of the Witpoort Formation of the Witteberg Group and is dominated by Rock, Cartref (soil depth 0.3–0.5 m; 10%–20% clay content) and Mispah soil forms (Visser et al. 1989, Land Type Survey Staff 2004). The plant communities at the KwaZulu-Natal localities also have well-drained rocky/sandy soil, with the Mispah and Glenrosa soil forms often recorded (Soil Classification Working Group 2001, Rutherford et al. 2006).

Both host plant species, *E. caffer* at Kabouga, and *S. eriopus* at Entumeni, are small cycads with subterranean stems and deciduous or semideciduous leaves (Whitelock 2002). *Encephalartos caffer* is listed as near threatened (A2cd; B2ab (iii,v)), and *Stangeria eriopus* is listed as vulnerable (A2acd + 4acd), both having a declining population size (Bösenberg 2022a, 2022b). However, the *E. caffer* population of Kabouga is considered healthy, with adult and juvenile plants as well as seedlings recorded during a recent survey (Bezuidenhout and Erusan 2018). *Encephalartos caffer* is sometimes found growing among quarzitic sandstone rocky outcrops (Bezuidenhout 2017). This may be the result of the protection offered by the surrounding rocks against the effects of natural veld fires, associated with the Zuurberg mountain range, on young plants, although it was noted that they grow more effectively after fire incident in the closed Quarzitic grassy Fynbos plant community.

*Encephalartos lehmannii* occurs within 5 km of the *E. caffer* population in Kabouga and *E. longifolius* within 20 km (Fig. 2c). *Encephalartos lehmannii* and *E. longifolius* mostly occur at low altitudes in Kabouga Thicket and Shrubland (Edwards 1983, Van Wyk et al. 1988, Hoare et al. 2006). These areas receive much lower rainfall and are characterized by short Kabouga thickets (Edwards 1983).

### Incidence of Larvae and Herbivory in Kabouga

We recorded *C. millari* for the first time during surveys of arthropod diversity associated with *Encephalartos* species in AENP in July 2022 (Janse van Rensburg et al. 2022). During these initial surveys, we visited approximately 200 *E. longifolius*, 50 *E. lehmannii*, and 50 *E. caffer* plants (Janse van Rensburg et al. 2022). *Callioratis millari* was not found on *E. longifolius* or *E. lehmannii*, although the presence of *Z. lepida* on these plants was confirmed (Janse van Rensburg et al. 2022). The incidence of herbivory was not recorded during the initial surveys.

Between 26 and 29 June 2023, we conducted further surveys of the cycads in the Kabouga and the adjacent Darlington sections of AENP. *Encephalartos caffer* population occurs in a relatively small area (3.9 ha), and we surveyed as many cycad individuals as possible. The *E. longifolius* and *E. lehmannii* populations occur in a large area, and our efforts focused on surveying individuals that were not surveyed in 2022. We visited 142 *E. caffer* plants (estimated 50% of the population), but unfortunately due to bad weather, we could only visit 34 *E. lehmannii* and 33 *E. longifolius* plants. Surveys of each individual plant involved a 5-min visual inspection of the top and bottom of each leaf to identify eggs, larvae, and signs of herbivory. The number of eggs and larvae was recorded, distinguishing between *Z. lepida* and *C. millari* based on descriptions of Staude (2001) and Staude and Sihvonen (2014). We recorded the incidence of herbivory (percentage of cycad individuals exhibiting signs of herbivory) and indicated when individuals were severely damaged (>50% of leaf area removed). Since no other herbivores were recorded feeding on the cycad leaves, all observed herbivory was attributed to the 2 moth species.

### Impact of the Apparency of Cycad Individuals on the Incidence of Herbivory

We also asked whether the apparency of cycad individuals affected the incidence of herbivory. The apparency of cycad individuals can be influenced by various factors, such as the plant size and density of other plant species covering individual cycads (Janse van Rensburg et al. 2023b). *Encephalartos caffer* individuals were categorized, based on size, into 3 groups: large adult plants (>6 large leaves), medium-sized plants and juveniles (<6 large leaves), and seedlings (2–3 small leaves). Similarly, *E. lehmannii* and *E. longifolius* individuals were classified as large (large multistemmed plants), medium (single-stemmed plants), and small (stemless seedlings and juveniles). The canopy cover of the other plant species was scored as dense (completely canopy-covered with foliage from other plants), intermediate (partially canopy-covered), and sparse (no canopy cover from surrounding plant species) (Edwards 1983). We used chi-square analysis to compare the incidence of herbivory between different individual sizes and levels of canopy coverage provided to cycad individuals by the associated plant species.

## Results

### Incidence of Larvae and Herbivory in Kabouga

In accordance with the initial surveys (Janse van Rensburg et al. 2022), no eggs or larvae from *C. millari* were observed on *E. longifolius* or *E. lehmannii* individuals. One *Z. lepida* egg cluster, containing 258 eggs, was recorded on *E. lehmannii. Zerenopsis lepida* eggs are easy to distinguish from *C. millari*, which has larger eggs and occurs in small egg clusters with up to 7 eggs (Staude 2001).

*Callioratis millari* larvae were recorded on 25.35% of the inspected *E. caffer* individuals (Table 1). Larvae mostly occurred solitarily. Of the *E. caffer* individuals with larvae present, the mean was 1.64 larvae per cycad individual, with 71.43% cycad individuals having only one larva (Table 1). In rare cases, up to 7 larvae were recorded on an individual plant, although they occurred on separate leaves, and no gregarious behavior was observed. Based on the descriptions provided by Staude (2001), most of the recorded larvae were in the fifth and sixth (final) instars. Larger larvae chewed on leaf edges, while smaller instars scraped the leaf surfaces, often causing the surrounding leaflet area to die and turn brown. No *E. caffer* individuals with new, soft leaves were observed, and all *C. millari* larvae appeared capable of consuming the older, tougher leaves. We also did not find any larvae feeding on plant species other than *E. caffer.*

**Table 1.** Incidence of *Callioratis millari* and *Zerenopsis lepida* egg clusters and larvae per cycad individual and incidence of leaf herbivory

Moth species	Host species	Incidence (%) of egg clusters	Incidence (%) of larvae	Total number of larvae	Mean number of larvae per cycad (of the cycads with larvae present)	Incidence (%) of herbivory	Incidence (%) of severe herbivory (>50% leaf area removed)
*Callioratis millari*	*E. caffer* (*n* = 142)	0	25.35	59	1.64	76.06	2.81
*Zerenopsis lepida*	*E. lehmannii* (*n* = 34)	3.03%	0	0	0	44.12	14.71
	*E. longifolius* (*n* = 33)	0	0	0	0	34.38	6.06

Many of the *E. caffer* individuals (76.06%) showed some signs of herbivory by *C. millari*. However, only 2.81% of the individuals showed signs of severe herbivory, whereby more than 50% of a leaf flush was destroyed. The general incidence of *E. longifolius* and *E. lehmannii* cycad individuals that exhibited herbivory by *Z. lepida* was lower (<50%) than that recorded for *C. millari* on *E. caffer* individuals, but the incidence of severely damaged individuals of *E. lehmannii* and *E. longifolius* was higher than those of *E. caffer.*

### Impact of the Apparency of Cycad Individuals on the Incidence of Herbivory

We found no significant difference between the proportion of each size class covered by dense, intermediate, or sparse vegetation (χ^2^ = 7.511, *P* = 0.111), suggesting that smaller cycad individuals were not more likely to be covered by other vegetation. The proportion of cycad individuals with signs of leaf herbivory increased with increasing plant size for both *C. millari* (χ^2^ = 19.465, *P* < 0.001) and *Z. lepida* (χ^2^ = 7.775, *P* = 0.021) (Table 2). *Encephalartos caffer* individuals that were overgrown by other plants had a significantly lower incidence of leaf herbivory by *Callioratis millari* (χ^2^ = 13.766, *P* < 0.001), whereas no significant effect was observed for *Z. lepida* on *E. longifolius* and *E. lehmannii* individuals (χ^2^ = 2.577, *P* = 0.276). Instances of *E. lehmannii* or *E. longifolius* individuals being entirely covered by surrounding vegetation were infrequent due to their large size.

**Table 2.** Percentage of cycad individuals from each size and canopy cover category showing signs of herbivory. Data for *Encephalartos lehmannii* and *E. longifolius* individuals were pooled because of the small sample size for the 2 cycad species and because they are impacted by the same moth species

Moth species	Host species	Plant size				Density of surrounding vegetation			
		Small	Inter	Large	Chi-square	Sparse	Inter	Dense	Chi-square
*Callioratis millari*	*E. caffer*	33.3	60.0	82.9	χ^2^ = 19.465, *P* < 0.001	79.5	37.5	0	χ^2^ = 13.766, *P* = 0.001
*Zerenopsis lepida*	*E. lehmannii*, *E. longifolius*	18.2	42.9	58.3	χ^2^ = 7.775, *P* = 0.021	44.7	35.3	0	χ^2^ = 2.577, *P* = 0.276

## Discussion

### Notes on the Phenology and Life History of *C. millari*

A detailed comparison of *C. millari* density between Kabouga and Entumeni is challenging due to the completion of only 1 yr of surveys in Kabouga and published sources on Entumeni lack detailed information. However, with the available data, the larval density in Kabouga appears comparable to previously recorded counts during egg and larval surveys of *C. millari* in Entumeni, which equates to approximately 1 egg or larva for every 3 cycad individuals surveyed (Louw and Armstrong 2018).

The flight period also seems similar between Kabouga and Entumeni. *Callioratis millari* appears to be univoltine, and its flight period in Entumeni was previously reported to start in mid-April, lasting for approximately 3 wk (Staude 2001). Larval stages take approximately 2.5 months to complete (Staude 2001). Our surveys in Kabouga were conducted by the end of June when most of the larvae were in their last instars. Therefore, it would appear that the period of *C. millari* moth activity in Kabouga starts by the end of March or early April, similar to what was reported in Entumeni. However, this would need to be confirmed through surveys during that period. This would mean that the flight period of *C. millari* coincides with the latter part of the season when rainfall and temperatures are lower (around April for both localities) and that the peak period of larval occurrence is during the driest and coldest months of the year (May–July). This flight period is similar to the univoltine *C. mayeri* Staude, which feeds on the cycad *E. friderici-guilielmi* Lehm., also in the Eastern Cape province (Staude 2001). However, it differs from the majority of other Diptychini species that feed on cycads since they are typically bivoltine or multivoltine, being present for more extended periods of the year (Staude 2001, Staude and Sihvonen 2014).

Staude (2001) did not record final-instar larvae of *C. millari* on *S. eriopus* in Entumeni and indicated that *C. millari* may have a secondary host plant, similar to other Diptychini, and that secondary host plant requirements could contribute to the species’ rarity. Supporting this, Staude (2001) found that final-instar larvae of *C. millari* accepted leaves of *Diospyros lycioides* Desf. (Ebenaceae) and flower petals of *Tropaeolum majus* L. (Tropaeolaceae) in captivity. It is worth mentioning that *D. lycioides* is a secondary host plant for several Diptychini species (Staude and Sihvonen 2014). However, in Kabouga, we found the final-instar larvae only on *E. caffer* individuals and not on any of the surrounding plant species. These neighboring species include various forbs such as *Erica adunca* Benth., *E. demissa* Klotzsch ex Benth., *E. pectinifolia* Salisb., *Gnidia anthylloides* (L.f.) Gilg, *Oedera imbricata* Lam., *Metalasia muricata* (L.) D. Don, *Podalyria burchellii* DC., *Leucospermum cuneiforme* (Burm.f.) Rourke, and *Leacadendron salignum* P.J. Bergius. Among the grasses noted were *Themeda triandra* Forssk., *Diheteropogon filifolius* (Nees) Clayton, *Tristachya leucothrix* Trin. ex Nees, *Eragrostis curvula* (Schrad.) Nees, *E. capensis* (Thunb.) Trin., *Trachypogon spicatus* (L.f.) Kuntze, *Sporobolus centrifugus* (Trin.) Nees, and *Alloteropsis semialata* (R.Br.) Hitchc. Additionally, the restioid *Restio triticeus* Rottb. and sedges like *Tetraria cuspidata* (Rottb.) C.B.Clarke and *Ficinia* Schrad. species were noted (Van Wyk et al. 1988). *Callioratis millari* may not have secondary host plant species in Kabouga, thus completing its entire life cycle on its cycad host plant. We also observed that larvae easily consumed the old leaves of *E. caffer*, although further confirmation is needed to determine whether the first instars could also consume old leaves.

We also investigated the relationship between the apparency of cycad individuals and herbivory. We found that more apparent individuals (individuals that are larger and covered by sparse vegetation) had a higher incidence of herbivory. Observations at Entumeni also indicated that *C. millari* avoided individuals growing under the Forest canopy, despite the higher abundance of cycad individuals under the Forest canopy cover (Staude 2001). *Callioratis millari* may be sensitive to factors like shading from canopy cover or increased foliar density that impedes larval movement. Other Diptychini species have also been demonstrated to have a higher affinity for larger cycad plant species or individual cycads that are sparsely covered by other vegetation (Bayliss et al. 2009, Janse van Rensburg et al. 2023b). This could be the case for the majority of Diptychini since they are diurnal moths, which most likely use visual cues in addition to cycad plant volatiles to locate host plants. Exceptions include *C. abraxas* Felder that prefer cycad individuals growing under a forest canopy and that are covered by dense foliage (Staude 2001).

### Habitat Requirements of *C. millari*

The cycad host plants of *C. millari* have a much wider occurrence, suggesting that host plant constraints are unlikely the main cause of the rarity of *C. millari.* With 2 known localities and the historic type locality, it was possible to make some initial comparisons. The habitats are associated with different plant communities, but there are some key similarities. All habitats have an open woody canopy cover within a grassy habitat, surrounded by patches of Forest. The open woody canopy cover could play a significant role in *C. millari* movement and host finding. While lek mating behavior has not yet been observed for *C. millari*, it is worth noting that several Diptychini exhibit this behavior, and the adjacent Forest patches might be necessary for providing suitably tall trees for lek localities. All habitats also have a diverse range of flowering plants that could serve as nectar sources for adult moths. Although the rainfall at Kabouga is lower than that at the other localities, the rainfall at this site is not low (>400 mm). Also, within Kabouga, *C. millari* and its host were only recorded on south-facing upper midslopes, which is wetter than those facing north due to morning dew persisting until noon on the south-facing midslopes. Due to this, grassy Fynbos generally occur on the south-facing upper midslopes at this locality, while the north-facing upper midslopes become Grassland (Van Wyk et al. 1988, Rebelo et al. 2006).

Although healthy populations of *E. lehmannii* and *E. longifolius* occur within 10 km of the *E. caffer* population, we did not record *C. millari* on these cycad individuals. Most of these individuals occur in a habitat that contrasts with that of the *E. caffer* population, consisting of Kabouga Thicket and Kabouga Shrubland at lower altitudes and with lower rainfall (Bezuidenhout 2015). The Kabouga Thicket and closed Kabouga Shrubland vegetation structure of the *E. lehmannii* and *E. longifolius* populations may be unsuitable for *C. millari.* However, many of the *E. longifolius* individuals also occur at similar altitudes as the *E. caffer* population, although it is associated with warmer northern facing steep upper midslopes of the Zuurberg mountain range. The quartzite outcrop habitat is dominated by a sparse Woodland of large individuals of *E. longifolius* with fewer grasses and more forbs species than described for the *E. caffer* locality of the Quartzite grassy Fynbos plant community. Furthermore, the physical leaf traits of *E. longifolius* may pose a challenge for early instar *C. millari* larvae. The young leaves of *E. longifolius* are finely tomentose, and the old leaves become very tough and leathery. It is uncertain whether *C. millari* larvae can consume these leaves, and this would need to be confirmed with rearing experiments. *Encephalartos caffer* individuals have small leaves and leaflets that are easily consumed by the mature larvae of *C. millari*. This may be an important requirement for *C. millari* since we did not find any secondary host plant species. Both *E. caffer* and *S. eriopus* are reported to be deciduous or semi-deciduous (Whitelock 2002) and possibly do not invest as much in leaf toughness as a result. The availability of new leaves is not always abundant, and without secondary host plant species, the food supply of *C. millari* may be too limited in cycad species that have old leaves that are too tough for *C. millari* to consume.

In addition to deciduous leaves, both *E. caffer* and *S. eriopus* have subterranean stems (Whitelock 2002). This may further indicate similarities between the habitats of *C. millari* in Kabouga and Entumeni. Cycads with deciduous leaves are typically found in grassy habitats that experience frequent fires (Whitelock 2002). During the summer months, thunderstorms are common in Kabouga, and the accumulated moribund biomass may cause fires (Van Wyk et al. 1988). Several studies have shown the importance of fire for Lepidoptera conservation (e.g., Vogel et al. 2007, Gaigher et al. 2018). Similarly, the natural fire cycle will likely have a very important role in maintaining the habitat of *C. millari* by preventing the buildup of woody vegetation, especially considering *C. millari* lays eggs on cycad individuals in open areas without canopy cover. Furthermore, fire may benefit the host plant of *C. millari* by stimulating the production of new leaves and cones (Cousins and Witkowski 2017).

However, burning at the wrong time of the year may have potential negative consequences. Diapausing pupae below the soil may be protected from lightning fires that occur during the summer months. Since the larvae of *C. millari* are present during the winter months, they may avoid fire; however, they could be adversely affected if fire occurs between April and July. Similarly, reports have indicated that indiscriminate fires during May adversely affected *C. millari* in Entumeni (Louw and Armstrong 2018). To prevent negative impacts on *C. millari*, it is suggested that if the burning of the Entumeni localities is required, these events should take place late in the season (end of August or September) (Louw and Armstrong 2018).

### Competition Between *Z. lepida* and *C. millari* in Kabouga

Although we have not observed *Z. lepida* on *E. caffer* individuals, it is known to be one of its host plants (Donaldson and Bösenberg 1995). It is, therefore, unlikely that the population of *E. caffer* in Kabouga is an unsuitable host population for *Z. lepida.* It is more likely that the *Z. lepida* population within Kabouga reaches its peak periods of activity at different times of the year, as we also only managed to record a single *Z. lepida* egg cluster in the *E. lehmannii* and *E. longifolius* populations during this period. Although *Z. lepida* is multivoltine, Staude and Sihvonen (2014) indicated 2 peak flight periods of *Z. lepida* in mid-summer and autumn, and records in June and July are generally scarce. *Zerenopsis lepida* activity is also generally absent from habitats that are colder and prone to frost during winter months (Janse van Rensburg et al. 2023b). Differences in peak flight periods and periods of larval activity between *Z. lepida* and *C. millari* indicate that there may be little competition between these 2 species in Kabouga as they avoid a temporal overlap. However, Entumeni, which experiences less frost, may have a greater abundance of *Z. lepida* in the winter months than Kabouga. This hypothesis, however, needs to be tested.

## Conclusions

The discovery of a new locality and host has significant implications for the ecology and conservation of *C. millari*:

1. The discovery of *C. millari* 750 km from the previously known localities indicates that its range is wider than previously thought. Despite extensive searches in KwaZulu-Natal, additional *C. millari* populations have not been located since the discovery of the Entumeni locality in 1997 (Staude 2001). *Encephalartos caffer* is a fairly common cycad species with numerous subpopulations in the Eastern Cape and an isolated subpopulation in KwaZulu-Natal. The distribution of *S. eriopus* also extends into the Eastern Cape. Therefore, further surveys in the Eastern Cape will be crucial since the presence of *C. millari* in other localities in this province may have been overlooked. Identifying other populations will allow a better comparison of habitats and a better understanding of habitat requirements.
2. The discovery of the new locality and host record adds to our understanding of its biology and provides valuable information on the ecological requirements as well as ecological preferences of *C. millari* and may ultimately contribute to informed management decisions.
3. The discovery of the new locality may also impact the conservation status of *C. millari*. It seems to be thriving in the newfound Kabouga locality; however, it is a small area and is vulnerable to stochastic events like untimely fires. Furthermore, given the ecological requirements of *C. millari* and the threat of poaching of their cycad host plants, this species warrants classification as highly threatened. Fortunately, Kabouga falls within a protected area, enhancing the conservation prospects of *C. millari*. The discovery of a rare moth within the AENP also adds to the conservation value of the national park. For conservation purposes, it is necessary to carefully monitor and protect the areas of occurrence.
4. This discovery presents an opportunity for further research, including the investigation of factors contributing to the rarity of *C. millari* and its life history to aid management decisions. It may also be important to establish the phylogenetic relationship between the Kabouga and Entumeni populations of *C. millari*, as well as investigate the host specificity of both populations. Staude (2001) determined, in rearing experiments, that the *C. millari* population in Entumeni accepts *E. villosus* leaves in captivity. Thus, it may potentially accept *E. caffer* as host, and the Kabouga population potentially may accept *S. eriopus* as host. Alternatively, both may exhibit host specialization at a population level and only accept hosts from their respective areas, which could suggest the presence of cryptic species. This possibility warrants further investigation.

## Supplementary Material

Supplementary material is available at *Environmental Entomology* online.

nvae008_suppl_Supplementary_Tables_A1
