# Supplementary material for: A new distribution and host record for the rare moth, Callioratis millari (Lepidoptera: Geometridae), and some ecological observations
Source: Environ Entomol. 2024 Feb 10;53(2):305–12. doi: 10.1093/ee/nvae008 (PMC11008734; doi:10.1093/ee/nvae008)
Supplement: nvae008_suppl_Supplementary_Tables_A1 [file nvae008_suppl_supplementary_tables_a1.docx]

**Table A1** Desktop comparison between the habitat of the current localities, the historical type-locality of *Callioratis millari,* and the localities in Kabouga from where *C. millari* is absent. Habitat characteristics derived from Van Wyk et al. (1988); Staude (2001); Rebelo et al. (2006), Rutherford et al. (2006), Hoare et al. (2006) and Louw and Armstrong (2018)

| **Habitat characteristics** | **Kabouga** | **Entumeni** | **Historical type locality** | **Kabouga, Darlington, Zuurberg** |
| --- | --- | --- | --- | --- |
| *C. millari* status | Present | Present | Extinct | Absent |
| Cycad host plant | *Encephalartos caffer* | *Stangeria eriopus* | *Stangeria eriopus* | *E. lehmannii, E. longifolius* |
| Altitude (m a.s.l.) | 950 | 700 | 700 | 250-600 |
| Plant community | Quarzitic grassy Fynbos | Moist Coast Hinterland Grassland | KwaZulu-Natal Sandstone Sourveld | Kabouga thicket and - shrubland |
| MAP^1^ (mm) | 544 | 888 | 934 | 334 |
| MAT^2^ (ºC) | 16.5 | 17.7 | 17.2 | 17.5 |
| Mean frost days | 5 | 2 | 2 | 8 |
| Habitat description | Steep south-facing mid-slope supporting grassy Fynbos with localised patches of dense proteoid and ericaceous Fynbos. | Dense tall grassland dominated by Ngongoni grass (*Aristida junciformis*) and *Cymbopogon nardus* in Entumeni. | Flat plateau tops with species-rich grassland and scattered low shrubs. Proteaceae trees and shrubs can be locally common. | Low mountains and foothills are covered with Thickets, where trees, shrubs and succulents are common. |
| Geology | White quarzitic sandstone and subordinate shale of the Witpoort Formation of the Witteberg Group and the rock-soil complex dominated by Rock, Cartref and Mispah soil forms | Acid, leached, heavy soils are derived from Karoo supergroup sediments and intrusive dolerites. Glenrosa and Mispah soils also occur | Nutrient-poor, skeletal, sandy soils freely drained and including Glenrosa and Mispah forms. | Soils are sandy and nutrient-poor and derived from the Bokkeveld and Witteberg groups. |
| Cycad characteristics | Small cycad with subterranean stems and deciduous to semideciduous leaves. | Small cycad with subterranean stems and deciduous to semideciduous leaves. | Small cycad with subterranean stems and deciduous to semideciduous leaves. | Large cycad with above-ground stems (up to 5 m in height), often multi-stemmed. Leaves are tough. |
| Area of occurrence | 3.9ha | <1 km^2^ | *-* | *-* |

1: Mean annual Precipitation; 2: Mean annual temperature.
